# Image-Based Method to Quantify Decellularization of Tissue Sections

**DOI:** 10.3390/ijms22168399

**Published:** 2021-08-05

**Authors:** Maria Narciso, Jorge Otero, Daniel Navajas, Ramon Farré, Isaac Almendros, Núria Gavara

**Affiliations:** 1 Unitat de Biofísica i Bioenginyeria, Facultat de Medicina i Ciències de la Salut, Universitat de Barcelona, 08036 Barcelona, Spain; mnarciso@ibecbarcelona.eu (M.N.); jorge.otero@ub.edu (J.O.); dnavajas@ub.edu (D.N.); rfarre@ub.edu (R.F.); isaac.almendros@ub.edu (I.A.); 2 The Institute for Bioengineering of Catalonia (IBEC), The Barcelona Institute of Science and Technology (BIST), 08028 Barcelona, Spain; 3 CIBER de Enfermedades Respiratorias, 28029 Madrid, Spain; 4 Institut d’Investigacions Biomèdiques August Pi i Sunyer, 08036 Barcelona, Spain

**Keywords:** segmentation, decellularization, microscopic image, fluorescence image, image processing

## Abstract

Tissue decellularization is typically assessed through absorbance-based DNA quantification after tissue digestion. This method has several disadvantages, namely its destructive nature and inadequacy in experimental situations where tissue is scarce. Here, we present an image processing algorithm for quantitative analysis of DNA content in (de)cellularized tissues as a faster, simpler and more comprehensive alternative. Our method uses local entropy measurements of a phase contrast image to create a mask, which is then applied to corresponding nuclei labelled (UV) images to extract average fluorescence intensities as an estimate of DNA content. The method can be used on native or decellularized tissue to quantify DNA content, thus allowing quantitative assessment of decellularization procedures. We confirm that our new method yields results in line with those obtained using the standard DNA quantification method and that it is successful for both lung and heart tissues. We are also able to accurately obtain a timeline of decreasing DNA content with increased incubation time with a decellularizing agent. Finally, the identified masks can also be applied to additional fluorescence images of immunostained proteins such as collagen or elastin, thus allowing further image-based tissue characterization.

## 1. Introduction

Tissue decellularization has become a very relevant method in recent years. On the one hand, the production of acellular scaffolds has applications in cell culture, tissue repair and regenerative medicine, while on the other hand, the study of the extracellular matrix (ECM) and its interactions with their homing cells can yield further understanding of multiple diseases and pathologies [1]. The ECM which remains after decellularization is composed of different proteins that provide structural support as well as physical and chemical signals to the cells that were embedded in it [2]. As such, it is important to guarantee that the ECM is preserved as much as possible while also assuring full cellular removal. Among other methods, the removal of cells from the ECM is often performed by a detergent, an enzymatic treatment, or a mix of both, followed by a DNA degradation step, usually benzonase or deoxyribonuclease (DNAse) [3].

To assess the decellularization of the tissue, the standard approach is to use absorbance-based DNA quantification [4,5,6,7]. This step can be performed with commercially available kits that typically work by digesting a piece of the decellularized tissue, isolating and purifying its DNA and using a spectrophotometer to measure the amount of DNA content per mg of dry tissue. While this method provides an initial quantitative estimate of DNA content, the tissue is conventionally classified as either successfully decellularized or not, depending on whether it passes the gold standard set by Crapo et al. in 2011 [8]: tissues are considered decellularized if their DNA content is below 50 ng per mg of dry tissue. In addition, cell nuclei can be visualized with a histological stain such as Hematoxylin and Eosin (H&E) [9,10,11] or by staining the DNA using 4’,6-Diamidino-2-Phenylindole, Dihydrochloride (DAPI) or Hoechst 33342 [10,12,13,14]. Optical microscopy images are then used to confirm the lack of visible nuclei when compared to the native tissue. The absence of or reduction in cell nuclei is usually measured in a qualitative manner, but some image processing tools such as ‘HisTOOLogy’ [15] have used the number of nuclei from H&E staining to quantitatively determine the decellularization of liver samples when compared to native tissue. Other decellularization assessment methods include the actual presentation of the organ, e.g., organ discoloration (from pink/red to white/transparent), as sufficient evidence of cellular removal [16]. Crapo et al. also suggested that the DNA fragment length should be below 200 bp; however, this metric is rarely assessed.

Even though these decellularization assessment methods are suitable for certain applications, they are not without drawbacks. ‘HisTOOLogy’, for instance, only takes into consideration the number of cell nuclei as a parameter for decellularization and cannot account for the DNA that has been released from the nucleus but is still present in the matrix. This measurement is extremely important especially for cell culture and tissue engineering applications, where DNA presence can be detrimental. DNA quantification kits, on the other hand, are time consuming and are preferentially suitable for full organ decellularization methods that provide large amounts of sample tissue. In the cases where decellularization is performed slice by slice, or biological material is scarce, such as in human biopsies, acquiring the minimum amount of tissue required to achieve a reliable readout may be prohibitive. In addition, DNA quantification kits require the digestion and consequent destruction of the probed tissue sample, an issue that further complicates matters in scarce samples. Finally, these kits are typically performed on a batch-by-batch basis to confirm the success of the decellularization protocol and assume the tissue is uniformly decellularized, disregarding inter- and intrasample variability considerations.

In this regard, it should be mentioned that biological tissue is heterogeneous. Taking the lung as an example, the alveoli and the pleura region have different cell and matrix distributions as well as different micromechanical properties [17,18]. These locations also have different components and structural organization of their ECM, which may cause the decellularization process to display heterogeneity across the tissue. This likely occurs when the aim of the decellularization process is to preserve and later visualize the 3D structure, composition, or mechanical properties of the ECM, and thus a milder agent is used. Therefore, there is a need for a minimally wasteful quantitative method that can reliably assess tissue decellularization via DNA quantification in a fast and simple manner. Accordingly, we propose an image-based approach that can quantify DNA content based on the combination of the DNA staining fluorescence image of cell nuclei and the phase contrast (PC) image of the (de)cellularized tissue.

Nuclear staining is extensively used for cell segmentation techniques [19,20], where the aim is to pinpoint nuclei or individual cells rather than to obtain quantitative measurements of their DNA content. However, in the decellularization protocol the nuclei are disrupted, causing the remaining DNA to diffuse throughout the tissue attaching itself to the remaining ECM. Additionally, DNA fluorescence intensity is expected to decrease and lose any distinct organization that can be easily segmented in decellularized (or partially decellularized) samples. Consequently, this fluorescence channel becomes unreliable for image segmentation strategies, raising the need for alternatives. As a solution, our novel method proposes the use of phase contrast images for tissue segmentation purposes only. Our protocol simply adds an extra step that can be readily incorporated in the standard acquisition of images of the UV fluorescence channel and any other immunohistochemistry fluorophores of interest. As such, the phase contrast image is used to segment a mask of the tissue of interest with respect to the naked glass substrate, whereas the fluorescence images are used for fluorescence intensity quantification purposes. We thus propose a novel method that offers quantitative information on the decellularization level of the sample and potentially also on the presence and organization of matrix proteins such as collagen or elastin, whilst using a minimal amount of biological tissue.

## 2. Results

**Figure 1:**
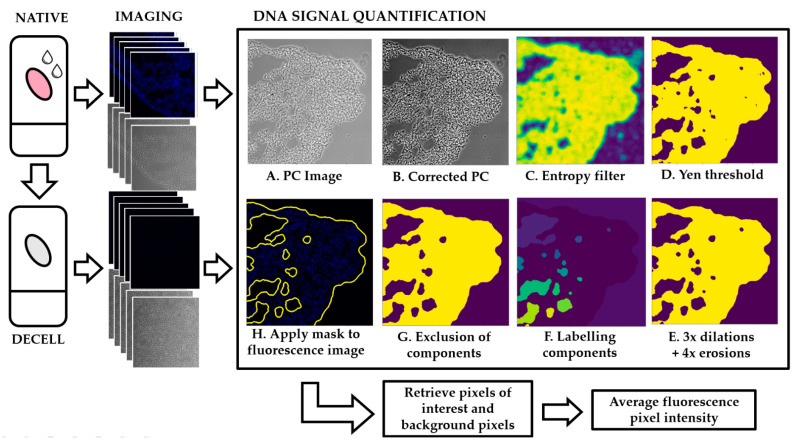
Schematic layout of the image processing pipeline. Native and decellularized samples are imaged and sets of image pairs (PC + UV) are taken. UV signal quantification: (**A**) the PC image is selected and (**B**) normalized for increased contrast. (**C**,**D**) An entropy filter is applied where high entropy regions correspond to high intensity regions and low entropy regions correspond to low intensity regions. (**E**) Three dilations followed by three erosions are applied to create smaller pixels aggregates and (**F**) these components are labelled. (**G**) Components are excluded/included in the mask depending on number of pixels. (**H**) The mask is applied to the UV image to identify pixels of interest corresponding to tissue vs. the background.

**Figure 2:**
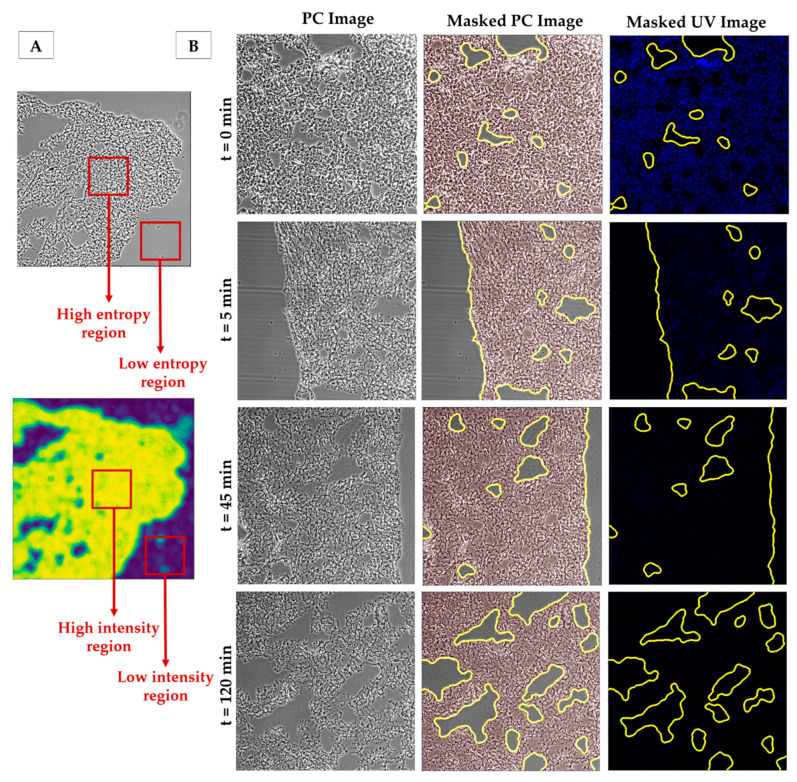
(**A**) Representation of the entropy-based segmentation: high entropy regions in the phase contrast image correspond to high intensity regions output image of the entropy filter. Low entropy regions corresponding to the background will result in low intensity regions in output of the entropy filter. (**B**) Examples of PC images (**left**), masks obtained using our algorithm superimposed on the corresponding PC image (**centre**) and their corresponding UV fluorescent images with the superimposed mask (**right**). Contrast of PC images has been increased here for the sake of readability. Images taken with a 10× objective.

The schematic layout of the image processing algorithm employed is illustrated in Figure 1. This algorithm segments PC images based on the entropy (or heterogeneity) of the tissue. Areas corresponding to tissue are expected to have high heterogeneity while areas corresponding to glass are expected to have low heterogeneity (represented in Figure 2A). Accordingly, an entropy filter is first run over the sample, and the resulting image is then thresholded to achieve a mask. Pixels from highly entropic areas (tissue) are included in a mask while pixels corresponding to the background are not. This mask is then applied to the UV fluorescence image, so that DNA signal intensity is measured only in the pixels that have been previously identified as belonging to tissue. Further details of each step can be found in the Materials and Methods section.

**Figure 3:**
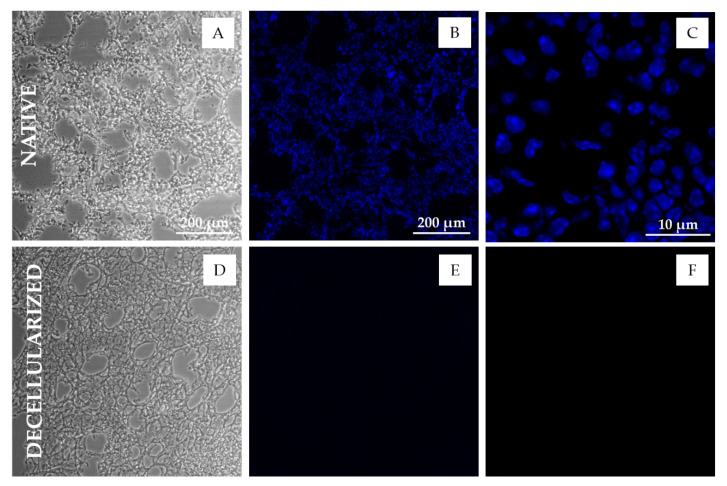
Microscopic images of lung tissue sections before and after decellularization. (**A**–**C**) PC image (**A**), DNA-stained fluorescent image (**B**) and confocal image (**C**) of native mice lung tissue sections (20 µm); (**D**–**F**) PC image (**D**), DNA-stained fluorescent image (**E**) and confocal image (**F**) of decellularized mice lung tissue sections (20 µm). Epifluorescent images were taken using a 10× objective. Confocal images were taken using a 60× oil immersion objective. DNA was stained by Hoechst 33342. Absence of nuclei and DNA is apparent in the decellularized sections (**E**,**F**).

Lung section decellularization with the standard decellularization protocol (SDC 2%) resulted in a clear DNA fluorescence signal reduction when compared to native sections (Figure 3). Images of decellularized samples showed no signs of visible cells in the tissue, while native sections showed distinct nuclei (Figure 3). Different levels of decellularization caused not only obvious differences in the DNA stained images of the tissues, but also changes in the morphology of the tissue resulting in clear changes in the PC images. In particular, with increased cellular removal, the average intensity of the PC images decreased noticeably. However, the algorithm was able to accurately perform segmentation on samples with different levels of decellularization (Figure 2B). The method was also able to correctly reject areas corresponding to tissue damage (Figure 2B), either caused by the cryosectioning process or the decellularization, as well as to correctly segment images with a reduced number of background pixels (Figure 2B (t = 0 min)).

As expected, decellularized lung sections had a significant reduction in DNA fluorescence intensity, displaying mean pixel fluorescence intensity values corresponding to only 5% of those displayed by native tissue. To validate the applicability of our method to tissues with a different ECM organization, we quantified samples of decellularized heart.

**Figure 4:**
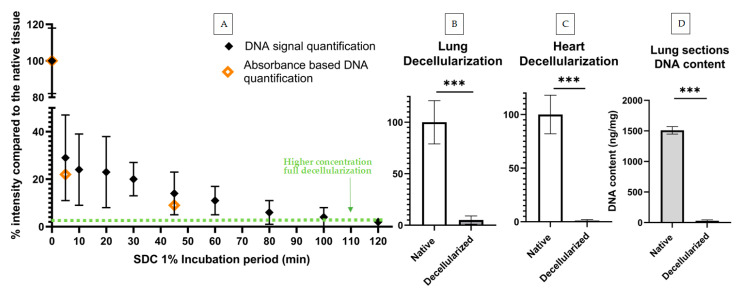
Lung and heart decellularization assessment via DNA signal quantification and DNA content quantification. (**A**) DNA signal quantification (*n* = 5, min of 8 sections per datapoint) and DNA content quantification (*n* = 1, a min. of 12 sections per datapoint) of decellularization protocols with increasing decellularization periods: results shown in % in comparison to the average DNA signal of the native tissue (100% intensity). Green dotted line corresponds to signal % of lung sections decellularized with standard concentration protocol (SDC 2%) (**B**) DNA signal comparison of native and decellularized lung sections (min. of 4 sections per datapoint) (**C**) and heart sections (min. 4 sections per datapoint). (**D**) DNA content of native and decellularized lung sections obtained by UV-absorbance DNA quantification (min. 12 sections per datapoint). Statistical significance between native and decellularized groups was verified using unpaired *t*-tests, *** indicates *p* < 0.001. DNA signal quantification of decellularization protocols with increasing decellularization periods: Results shown in % in comparison to the average DNA signal of the native tissue (100% intensity). All timepoints showed significant statistical differences (*p* < 0.05) versus native tissue (here shown as t = 0 min). A minimum of 10 image sets were taken for each lung section.

Of note, the same algorithm was used to segment heart section PC images without alterations and was able to produce consistent results. Similarly to lung, Median Set Fluorescence intensity (MSFI) from decellularized heart sections was only 2% of the native heart tissue MSFI (Figure 4C).

We compared the previous lung decellularization results to standard methods to quantify DNA content, in particular a DNA quantification UV absorbance kit. For this, we used 30 consecutive lung tissue sections, amounting to approximately 8 mg. Half of these sections were decellularized with the standard procedure and half were used as native controls. Following the decellularization step, tissue sections were scraped off from their substrates and pooled together in two groups (decellularized vs. native) and the DNA quantification was carried out according to the manufacturer’s instructions. We found that DNA present in decellularized slices was 2% of the total DNA present in native sections, showing good agreement with our results based on image quantification (Figure 4B).

The previous results were based on leaving the decellularization agent for 30 min, as is standard protocol in our laboratory for this sample type and thickness to achieve full decellularization. Nevertheless, we hypothesized that the level of decellularization and cell removal is time-dependant and thus aimed to obtain its timeline in order to further verify our method. By decreasing the concentration of the decellularizing agent by half (from SDC 2% to 1%) and incubating the samples for different timepoints with this agent, we were able to quantify a broader range of DNA content instead of full decellularization only. Samples were incubated with SDC 1% for different time periods: 5, 10, 20, 30, 45, 60, 80, 100 and 120 min. Control samples were solely washed with phosphate-buffered saline (PBS) to remove OCT compound. To decrease biological variability, all sections for each experimental repeat had been consecutively cryosectioned from the same lung. Two consecutive sections were placed side by side in a glass slide to further decrease variability. All time points were imaged and the DNA signal quantified following our fluorescence quantification method. For validation, a reduced number of experiments was carried out using the standard UV-absorbance based method. In particular, DNA content was assessed for the decellularization protocols corresponding to timepoints t = 0, 5 and 45 min. Of note, UV-absorbance required processing together a minimum of 12–20 lung slices, depending on lung section area, (~20–30% of a mouse lung) to measure a single datapoint with minimally adequate signal-to-noise levels. We thus limited our validation to three timepoints to reduce the number of murine lungs that would have been otherwise required. As expected, the MSFI decreased with increasing SDC incubation time until it reached a plateau of 1% native intensity after the 80 min mark. The samples with the shortest incubation time already showed a significant decrease in MSFI when compared to native MSFI (Figure 4). All incubation periods produced statistically significant differences (*p* < 0.05) when compared to the untreated native samples. Standard deviation also sharply decreased with increasing SDC incubation periods, likely reflecting the homogenization of tissue as cells were increasingly eliminated from the tissue. Regarding DNA content assessed through UV-absorbance, the results from the tissues decellularized with the 5- and 45 min protocols were in line with the ones produced via signal quantification. As expected, both these protocols did not fall below the gold standard of 50 ng per mg dry weight: the 5 min protocol produced a concentration of 358 ng/mg and the 45 min protocol resulted in a concentration of 148 ng/mg.

To assess the precision and repeatability of the assays, we calculated the intra- and intersample variability of the native tissue’s MSFI by computing the coefficient of variation (CoV). Native measurements were chosen for this analysis over decellularized ones because the CoV would be artifactually large in decellularized samples since their MSFI is close to zero. Consecutive lung slices (2 slices per condition) were imaged from 5 independent experiments (*n* = 5) experiments—10 total samples. DNA was quantified with the above-described algorithm. Intrasample variability was obtained by averaging the CoV’s of each sample. Intersample variability was obtained by computing the CoV of the means of each sample. The results for intra- and intersample variability were very similar; 16.4% ± 9.0% and 17.9%, respectively. The similarity of these results suggests that the observed variability is mostly due to the naturally occurring biological variability of the tissue and not due to measurement or experimental error.

**Figure 5:**
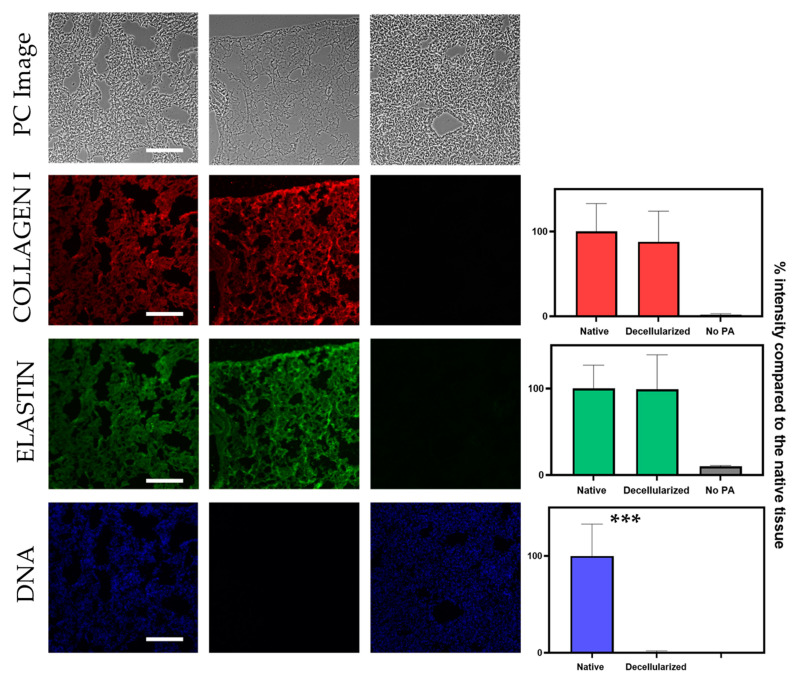
Simultaneous signal quantification of DNA, collagen and elastin signal based on segmentation of the corresponding PC image. The left column corresponds to a native lung section, the middle column corresponds to a decellularized lung section and the right column corresponds to a native section that was not treated with PA. Scale bar corresponds to 200 µm. The contrast of PC images has been increased for the sake of readability. Unpaired *t*-test for significance between native and decellularized groups with *p*-value *** *p* < 0.001. A minimum of 8 sections were used per datapoint.

In a decellularization protocol, cellular removal should be performed whilst also preserving the ECM proteins and structure as much as possible. Thus, the previously described algorithm was adapted to quantify the fluorescence signal from different channels corresponding to different matrix proteins: in this case, collagen type I and elastin (Figure 5). As expected, collagen and elastin signal in the decellularized samples showed no significant differences when compared to native sections. As expected, samples that were only incubated with the secondary antibody showed low levels of signal intensity: 2% and 10% for the channels corresponding to collagen and elastin, respectively. In line with the previous results shown, DNA signal decreased significantly in decellularized samples when compared to native sections. The intrasample variability of this analysis was performed as described before. Intrasample variability of the collagen and elastin experiments were very similar to the ones obtained for the DNA signal quantification: for the elastin channel, intrasample variability was 12.0% ± 5.0% and 15.6% ± 5.0% for the collagen channel.

## 3. Discussion

Overall, our image-based method was able to effectively mask tissue sections based on the PC images, in order to then extract the pixels of interest from the corresponding DNA stained images. The entropy-based segmentation was successful for both lung and heart decellularized tissue sections. Furthermore, the results achieved with the image-based approach were comparable to the UV-absorbance based results. As further proof of concept, we were able to track the decrease in DNA content with increasing tissue incubation periods with a decellularizing agent.

We have primarily focused our study on lung tissue and have thus taken into consideration its particular structure for some steps of the code. In particular, the lungs have different structures that need to be accounted for when performing image segmentation: alveoli, blood vessels and airways. Our code was written to include alveoli as part of the tissue and exclude the lumen of large blood vessels and airways. The reasoning for this decision is that the alveoli’s presence and size is consistent throughout lung tissue, but large blood vessels and airways are not and can influence significantly average pixel intensity.

In the future, this method could be expanded to provide additional information on the decellularization process. For example, to assess the structural integrity of the ECM, the same sample (or consecutive sections) could be imaged before and after decellularization and the free space area could be computed as a measure of tissue integrity. Additionally, by using higher resolution objectives paired with fluorescent staining, information about fibre density for native and decellularized tissue could also be obtained. Other applications include the study of ECM protein distribution, which can be especially relevant for conditions which involve the reorganization of the ECM, such as lung fibrosis [21] and lung cancer [22]. However, these additional studies were beyond the scope of this work.

Our method can also be applied to other decellularized tissues besides lung, since it is based on the natural morphological heterogeneity of the tissue, as shown by the heart decellularization dataset results. Other cell or tissue image segmentation methods are based on pixel intensity [23] but this technique could pose a challenge for thin sections such as the ones used in this work. Cryosectioning, or sectioning in general, can cause thickness variations across tissue slices that translate into different pixel intensities. Additionally, cellular removal naturally leads to a decrease in tissue density as can be seen in Figure 2, Figure 3 and Figure 5, which in turn leads to a decrease in intensity of the decellularized PC images. For this application, intensity-based segmentation would potentially require manual thresholding, fine tuning the upper- and lower-pixel intensity bounds for each experimental condition. By using tissue heterogeneity measured with an entropy filter, tissue thickness variation does not influence the segmentation step, allowing us to automate the thresholding steps and thus significantly decreasing the user input in the image analysis process. In addition, since this method is based on image entropy and not on directly thresholding intensity values, uneven illumination and other similar artifacts should not affect the accuracy of image segmentation. Tools such as the ones described in [15] rely on coloured images of non-fluorescent staining such as H&E. These tools offer a wide range of applications for quantitative analysis of histological sections; however, when applied to the problem of decellularization quantification, this method cannot solve the full problem since it only quantifies the number of nuclei present and not the DNA that may have been left behind by cellular lysis. Additionally, this method relies on a much more time consuming staining protocol, taking upwards of 24 h compared to the 40 min length of the nuclei-labelling protocol described previously.

The algorithm’s first steps leading to image thresholding are designed to be particularly stringent, initially rejecting some pixels of interest that may be later included in the mask. The following step then groups connected pixels into aggregates so they can be labelled and sorted according to their aggregate size (i.e., number of pixels per pixel aggregate). These pixel aggregates are then filtered in or out of the mask depending on size, allowing us to select biologically relevant structures. For example, in our imaging conditions of the lung, blood vessels correspond to components of >2000 pixels while alveoli are approximately 500 pixels. For other biological tissues, or when using other magnifications, these parameters can be tuned to include or exclude from the mask certain features that are considered biologically relevant. While we used a 10× magnification objective in our imaged samples, our protocol is also suitable for higher resolution objectives equipped with phase contrast rings. However, when using higher magnifications, we would recommend a larger dataset per sample (i.e., larger number of images per slice), with a minimum of 50% of the total sample area imaged.

When comparing both DNA quantification methods, the results were similar. The DNA levels in the decellularized samples were significantly reduced in comparison to native samples and well below the 50 ng dsDNA (double stranded DNA) per mg threshold established by Crapo et al. (2011) [8]. However, not only is DNA quantification from DNA extraction more costly and requires larger amounts of tissue, but it is also more time-consuming and typically destroys a large amount of the sample to yield only one metric. In this connection, our method is particularly convenient for samples where the full organ is not decellularized or the decellularization is carried out in thin tissue slices. For the specific decellularization protocol used in this work, we had to scrape off around 30 tissue sections from glass slides to obtain the minimal mass of dry tissue required for the DNA kit. While this approach was carried out here with the aim of validating our proposed method against an accepted gold standard, it would be difficult and time-consuming to obtain this amount of material in our experiments as routine, thus making the standard protocol for DNA quantification impractical and suboptimal. Similarly, our approach would be also best suited for clinical applications since it requires a minimal amount of biological tissue (two thin sections). For example, biopsies and other similar patient samples are typically scarce and should not be destroyed due to their uniqueness and the potential need for further characterization and testing. This new approach would thus enable easy and low-cost diagnostic applications in a clinical setting since it allows the decellularization of samples and quantitative confirmation of the procedure, followed by ECM characterization and any additional tests deemed necessary with the retained tissue.

Another major advantage of this method is that the potential multichannel image analysis allows for more than DNA content and nuclei presence assessment. In particular, it also allows researchers to gather information on the structural integrity of the matrix post decellularization, as well as its organization and composition. Other works have performed a semi-quantitative assessment of matrix proteins: for example, ECM proteins in different stages of osteogenic differentiation after decellularization were quantified using image analysis software (ImageJ and Adobe Photoshop) [14]. However, this quantification was performed with manual segmentation of areas of interest which does not allow for the analysis of large datasets or a great number of different experimental conditions, which would provide a more comprehensive understanding of the effects of the decellularization process on the ECM. For our work, we tested collagen and elastin as common ECM proteins but other matrix proteins, such as laminin and proteoglycans, could also be used. In addition, further characterization techniques could have been used such as second harmonic imaging microscopy or AFM-based mechanical testing.

Finally, most current fluorescence microscopes incorporate high degrees of automation, motorization and autofocus, thus allowing the seamless acquisition of hundreds of phase contrast + fluorescence image pairs covering the entire surface of a tissue of interest in a matter of minutes and with little human intervention. As such, our method is built into an image analysis pipeline, which allows for the accurate and unbiased analysis of dozens of stored images in minutes, even from different experimental conditions or samples. Accordingly, the combination of automated image acquisition and image quantification analysis constitutes a timely and promising new avenue for approaches that have traditionally relied on absorbance-based methods such as DNA quantification.

## 4. Materials and Methods

### 4.1. Sample Preparation and Decellularization

Lungs and hearts were harvested from adult mice, embedded in Optimum Cutting Temperature compound, OCT (Tissue-Tek, Sakura, Torrance, CA, USA) and stored at −80 °C. Samples were sectioned at 20 µm using a cryostat, with a working temperature of approximately −24 °C. Sections were deposited onto a positively charged glass slide (Superfrost; Thermo Fischer Scientific, Waltham, MA, USA) and dried for 15 min before being stored at −80 °C until further use.

Before decellularization, the edges of each slide were traced with a liquid repellent slide marker pen to keep liquid from spilling. Acellular sections were produced by consecutive washes and rinses of the sliced section while still firmly attached to a glass slide. To lyse the cells, the steps were as follows: tissue sections were twice incubated for 10 min with deionized water. Slices were then immersed in sodium deoxycholate (SDC) for a total of 30 min (2 consecutive 15 min wash). After removing the SDC with three 5 min PBS washes, tissue sections were incubated for 20 min in DNAse I. DNase was removed with the same washes described before. Sections were decellularized at a low agitation (80 rpm) in an orbital shaker to ensure even cellular content removal throughout the sections.

DNA of cellular and acellular samples was stained by incubation with NucBlue™ Live ReadyProbes™ Reagent (Hoechst 33342, Thermo Fisher Scientific, Waltham, MA, USA) for 20 min at 80 rpm in an orbital shaker followed by three 5 min PBS washes to remove excess staining with the same agitation settings. The Nucblue staining concentration was 1 drop per 500 µL of PBS, following the manufacturer’s instructions. Finally, samples were mounted in Fluoromount mounting media (Thermo Fisher Scientific) and stored at 4 °C.

### 4.2. Immunofluorescent and DNA Staining

For native sections, immunofluorescent staining was performed after removing OCT with consecutive washes of PBS. The tissue was fixed using 4% paraformaldehyde (PFA) for 30 min at room temperature. Samples were then blocked with 10% foetal bovine serum (FBS), and 1% bovine serum albumin (BSA) for 45 min at room temperature. Primary antibodies against elastin (goat anti-elastin, 1:50, Santa Cruz, Dallas, TX, USA) and against collagen I (rabbit anti-collagen I, 1:300, Abcam, Cambridge, MA, USA) were incubated in a solution of 10% FBS, and 1% bovine serum albumin (BSA) overnight at 4 °C at constant agitation (80 rpm). Slices were allowed to warm up at room temperature for 15 min and rinsed three times with the same type of solution. Secondary antibodies (goat anti-rabbit Alexa Fluor 647, 1:200 Thermo Fischer and donkey anti-goat, Alexa Fluor 488, 1:200, Thermo Fischer) were incubated at a 1:200 dilution in 10% FBS, and 1% BSA for 2 h, at 37 °C and constant agitation (80 rpm). Three 15 min rinses with PBS were applied to eliminate the unbound secondary antibodies. DNA of cellular and acellular samples was stained by incubation with NucBlue™ Live ReadyProbes™ Reagent with the same procedure described before. A few sections were subjected to the same protocol solely incubated with secondary antibodies to check for unspecific binding.

### 4.3. Experimental Setup and Protocol

Before imaging, glass slides were thoroughly cleaned with 70% ethanol and lint free tissue or lens tissue, to avoid artifacts in background detection. Epifluorescence images of the tissue sections were acquired with a Leica SP5 inverted microscope equipped with a CCD camera (C9100, Hamamatsu Photonics K.K., Hamamatsu, Japan) and using a 10× Plan Fluor objective (Nikon, Tokyo, Japan). For higher resolution images of the cell nuclei (or their absence), a Nikon D-Eclipse Ci confocal microscope was used in conjunction with a 60× Plan Apo immersion oil objective (Nikon).

Images belonging to a given treatment (decellularized slices) and corresponding control condition (native slices belonging to the same organ and animal) were acquired in a single imaging session. Exposure times for the PC and the fluorescent channels were set based on the control (native) sections corresponding to each experiment. These parameters were set by nearing pixel saturation as much as possible. In each location, PC and fluorescent images were acquired sequentially forming an image set and were saved as a single file with the file format.nd2. These image sets were 14bit 1000 × 1000 px images with 0.80 µm/px spatial resolution. Each channel was converted to 8 bit RGB .tif images by using the software NIS-Elements (Version 5.21.00, Nikon). Twenty locations per condition were chosen except when less than twenty locations were required to cover the complete slice. Slice locations that were visibly damaged were not imaged.

### 4.4. DNA Quantification from the PC and Fluorescence Image Pair

A custom Python image processing algorithm was developed to use PC images in order to quantify DNA presence in lung sections. This algorithm was implemented using mainly two Python libraries: skimage [24] and OpenCV [25]. In brief, the main goal was to use the PC image to identify the pixels that corresponded to tissue, thus creating masks for tissue and background. Subsequently, the masks were used to determine the fluorescence intensity of the DNA signal images corresponding to samples treated with decellularizing agents and comparing this value with the corresponding, untreated, native tissue.

The algorithm is based on three independent steps: (1) image pre-processing, (2) image segmentation and (3) mask refinement. In the image pre-processing stage, the input dataset is separated into image sets (PC image + fluorescent images). These images are transformed from RGB to greyscale. To improve image quality and contrast, the algorithm performs a contrast stretching, or normalization (Figure 1B. This step “stretches” the range of pixel intensity values to span the full range of pixel values allowed. Additionally, the tail ends of the picture histogram (below 2% and above 98%) were clipped to further improve image contrast.

For image segmentation, corrected PC images are subjected to an entropy filter (Figure 1C). The entropy filter returns the minimum number of bits needed to encode the local pixel intensity distribution and is computed using a base 2 logarithm. The structuring element used for this filter was a circle with radius of 5 pixels. The output of this step is an image where the higher entropy regions are brighter and low entropy regions are dimmer. Areas corresponding to matrix, either native or decellularized, have higher local variations in intensity throughout the tissue and therefore display larger entropy values. Areas corresponding to the background, which has constant intensity, will display lower values of entropy. The next step is to apply a threshold to this output based on Yen’s thresholding method (Figure 1D) [26]. High intensity pixels are selected and included in the mask while low intensity pixels will be excluded and dimmed as background.

The last step is mask refinement. To select which components should be included or excluded from the mask, we applied three dilations followed by four erosions (Figure 1E). For this operation, to separate components previously connected without decreasing the quality of the mask, a circular structuring element of radius three was determined to be the best approach. The number of erosions is greater than the number of dilations to combat the slight overestimation caused by the entropy filter. Each component was labelled and selected by number of pixels (Figure 1F,G). This number was chosen based on the biology of the tissue: components corresponding to large blood vessels, airways or tissue ruptures were excluded from the mask. The mask was inverted, and the same technique was applied to the background: regions caused by illumination artifacts or residue on the glass slide were thus eliminated.

The final mask is applied to the DNA signal image to obtain the pixels of interest (Figure 1H). The mask is then inverted and applied to the image again to extract the background pixels.

After selecting the pixels of interest, the mean is computed by summing the intensity of these pixels and dividing it by the number of pixels of interest. To that value, the median value of the background pixels is subtracted, as such:

(1) $$\mathrm{MFI}=\frac{\sum \mathrm{intensity}\;\mathrm{pixels}\;\mathrm{of}\;\mathrm{interest}}{\mathrm{number}\;\mathrm{of}\;\mathrm{pixels}\;\mathrm{of}\;\mathrm{interest}}-\mathrm{MedFBI}$$

where MFI is the mean DNA intensity and MedFBI is the median fluorescent background intensity, determined from the background pixels obtained by inverting the mask and applying it to the UV image.

The algorithm was written into a pipeline to be able to analyse a dataset of images at a time. This dataset corresponds to a specific condition (i.e., native, treated with decellularizing agent A, or B, etc.). The output of this algorithm is the MFI of each numbered image set and the corresponding background intensity. The median of this set of values-Median Set Fluorescence Intensity, *MSFI*-represents the fluorescence intensity corresponding to a specific treatment or condition.

To assess the decellularization level of a sample, this value is then compared to the median intensity value of the native samples of that same experiment, resulting in a final value which is a fraction of the native DNA intensity, as such:

(2) $$\mathrm{native}\;\mathrm{fluorescence}\;\mathrm{signal}\;\%=\frac{{\mathrm{MSFI}}_{\mathrm{decellularized}}-{\mathrm{MSFI}}_{\mathrm{native}}}{{\mathrm{MSFI}}_{\mathrm{native}}}\times 100\%$$

where MSFI is the median fluorescence intensity of a specific image dataset.

### 4.5. Multichannel Analysis

The same algorithm was adapted to quantify collagen and elastin content based on PC image masking and fluorescence signal quantification.

The process to generate a mask based on PC imaged was conducted as described before. In addition to quantifying DNA signal, the method was adjusted to also obtain information on the collagen and elastin content of both native and decellularized samples, a tool useful to not only quantify decellularization but also to assess the matrix conservation of decellularized samples. Collagen and elastin images were acquired simultaneously with UV and PC images, all corresponding to the same location.

In this specific protocol, instead of forming image pairs, the algorithm grouped images in sets of 4. When transforming the image from RGB to greyscale, different channels were selected depending on the fluorescent image: for DNA-stained images, values were taken solely from the blue channel, collagen from the red channel and elastin from the green channel. We decided on the following formula to transform RGB images to greyscale:

Greyscale = 0.299R + 0.587G + 0.114B(3)

The mask generated through the PC images was then applied to the 3 fluorescence images to obtain the pixels of interest for each one. Then, the mask is inverted and applied once again to the 3 fluorescence images to obtain the background pixels. MFI of each image was computed as described before.

### 4.6. DNA Quantification from DNA Extraction

The kit PureLink^®^ Genomic DNA Mini Kit (Thermo Fisher Scientific) was used to extract and quantify DNA. Briefly, this procedure digests tissue by employing Proteinase K to produce lysates which are subjected to several purifying steps. The yield of purified DNA can be estimated by UV absorbance at 260 nm using a Colibri LB 915 Microvolume Spectrophotometer (Berthold Technologies, Bad Wildbad, Germany).

To achieve enough tissue mass for the DNA quantification, native and decellularized 20 µm sections were retrieved from the glass slides by using a cell scraper and transferring the tissue onto an Eppendorf.

To compute the tissue mass, lung sections were modelled after an elliptic cylinder. The height of the cylinder was 20 µm and the cross sections were modelled as ellipses. Thus, the area of the cross sections was computed as

A_ellipse_ = π × R_1_ × R_2_ (4)

where R_1_ and R_2_ are the minor and the major radii, respectively. The volume was obtained by multiplying the area of the cross sections by the section thickness (or height, h):

(5) $$V_{\mathrm{lung}\;\mathrm{sections}}\;\approx {\;V}_{\mathrm{ellipse}}{=A}_{\mathrm{ellipse}}\times h$$

To compute the mass of each section, we used the density formula:

(6) $$\rho_{lung}=\frac{m}{V}$$

where ρ is the lung density, m is the mass and V is the volume. In accordance with the work of described in [27] we considered mice lung density to be 0.3 g/cm^3^. A total of 30 sections were used for cellular and acellular DNA quantification testing (15 for each condition). The lung samples tested were consecutive cryosections from the same lung.

By quantifying the DNA of both cellular and acellular lung samples, we were able to compute an approximate absolute value for DNA per mg of tissue for decellularized samples and also a relative value by comparing acellular to native tissue, which follows the same method as the image-based approach.

### 4.7. Statistical Analysis

All data obtained from various experiments followed a normal distribution. For experiments with 2 groups (native and decellularized) statistical comparisons were performed by an unpaired two-tailed t test. One-way analysis ANOVA with Tukey’s comparison test was used to determine statistical differences between the different groups subjected to different incubation periods compared to the untreated samples. All data are mean ± SD. Differences were considered statistically significant for *p* < 0.05. Statistical analysis was performed using GraphPad Prism (GraphPad software 9.1.0, Inc., San Diego, CA, USA).

Intra- and intersample variability were computed using the coefficient of variation (CoV), as follows:

(7) $$CoV=\frac{\sigma }{\mu }$$

where σ is the standard deviation and *µ* is the mean.

## Data Availability

The code developed for segmentation of phase contrast images will be made available at Github.
